# Improving the performance of 3D image model compression based on optimized DEFLATE algorithm

**DOI:** 10.1038/s41598-024-65539-7

**Published:** 2024-06-28

**Authors:** Xue Kai, Zhang Yuxiang

**Affiliations:** 1 School of Humanities and Social Science, Xi’an Jiaotong University, Xi’an, 710049 Shaanxi China; 2 Zhang Yuxiang School of Microelectronics, Xian, 710126 China

**Keywords:** DEFLATE algorithm, 3D image model compression, Compression ratio, Compression time, Performance optimization, Mathematics and computing, Computer science, Information technology

## Abstract

This study focuses on optimizing and designing the Delayed-Fix-Later Awaiting Transmission Encoding (DEFLATE) algorithm to enhance its compression performance and reduce the compression time for models, specifically in the context of compressing NX three-dimensional (3D) image models. The DEFLATE algorithm, a dual-compression technique combining the LZ77 algorithm and Huffman coding, is widely employed for compressing multimedia data and 3D models. Three 3D models of varying sizes are selected as subjects for experimentation. The Wavelet algorithm, C-Bone algorithm, and DEFLATE algorithm are utilized for compression, with subsequent analysis of the compression ratio and compression time. The experimental findings demonstrate the DEFLATE algorithm’s exceptional performance in compressing 3D image models. Notably, when compressing small and medium-sized 3D models, the DEFLATE algorithm exhibits significantly higher compression ratios compared to the Wavelet and C-Bone algorithms while also achieving shorter compression times. Compared to the Wavelet algorithm, the DEFLATE algorithm enhances the compression performance of 3D image models by 15% and boosts data throughput by 49%. While the compression ratio of the DEFLATE algorithm for large 3D models is comparable to that of the Wavelet and C-Bone algorithms, it notably reduces the actual compression time. Furthermore, the DEFLATE algorithm enhances data transmission reliability in NX 3D image model compression by 12.1% compared to the Wavelet algorithm. Therefore, the following conclusions are drawn: the DEFLATE algorithm serves as an excellent compression algorithm for 3D image models. It showcases significant advantages in compressing small and medium-sized models while remaining highly practical for compressing large 3D models. This study offers valuable insights for enhancing and optimizing the DEFLATE algorithm, and it serves as a valuable reference for future research on 3D image model compression.

## Introduction

Data compression has become a crucial challenge in the era of big data. The Delayed-Fix-Later Awaiting Transmission Encoding (DEFLATE) algorithm has emerged as a highly practical compression technique extensively employed in various domains, including image, text, and audio compression^1–3^. This study aims to assess the efficacy of the DEFLATE algorithm in compressing NX three-dimensional (3D) image models while optimizing the required storage space and transmission time, thereby enhancing transmission efficiency and data storage performance. The significance of exploring the notable advantages of the DEFLATE algorithm in this field is underscored^4,5^. Buccino et al.^6^ proposed a fracture mechanics-based method for quantifying critical stress enhancement in healthy and osteoporotic trabecular bone using synchrotron scanning combined with micro-mechanical testing. This method is complemented by a morphological and densitometric framework to capture differences in pore networks when pathological changes are present. To address the current time-consuming and computationally expensive manual/semi-automatic segmentation steps, they implemented convolutional neural networks (CNNs) to detect the initiation and propagation of microscale damage. The results highlight the close interaction between toughening and weakening phenomena at the microscale as fundamental aspects for preventing fractures. Goff et al.^7^ emphasized the importance of post-processing synchrotron data, particularly in understanding the relationship between bone cell lacunar morphology and disease. In a subsequent study, Buccino et al.^8^ analyzed bone cell lacunae in large-scale synchrotron image-guided skeletal failure assessments, demonstrating the significance of synchrotron imaging in studying early signs of bone pathology. They also underscored the value of using artificial intelligence tools for data analysis, providing additional information and insights for orthopedic clinical practice.

In the realm of compressing NX 3D image models, the inevitable issues of transmission time and storage space necessitate the application of compression algorithms^9^. These challenges impede the efficient transmission and utilization of NX 3D image model data. This study addresses the inherent redundancies in 3D model data and continually enhances and optimizes the DEFLATE algorithm to simultaneously improve the compression ratio and decompression speed. Furthermore, it evaluates the superiority of the DEFLATE algorithm compared to other compression algorithms. By effectively reducing the space and transmission time occupied by 3D model data, the utilization of the DEFLATE algorithm contributes to improved transmission efficiency and data storage performance.

Based on the specific compression requirements of the NX 3D image model, this study employs optimization and design techniques such as compression tables, dynamic Huffman coding, and bitstream composition to enhance the existing DEFLATE algorithm. The objective is to propose an optimized DEFLATE algorithm workflow to meet the compression needs of the NX 3D image model. Different compression algorithms and parameters are utilized for compressing images at varying levels. The compression time is reduced by minimizing the encoding of infrequently occurring characters. This study holds practical reference value for optimizing the compression of 3D image models and establishing a scientific and standardized process.

## Related works

### Recent advances in image compression algorithms and the DEFLATE algorithm

In the context of modern medical imaging technology, image processing and analysis have become integral to the diagnosis and treatment process. The integration of artificial intelligence and swarm intelligence algorithms has enhanced the accuracy and efficiency of medical image processing, thereby providing reliable support for clinical diagnosis and treatment. Hu et al.^10^ proposed a fuzzy systems-based medical image-processing method for brain disease prediction. By leveraging the uncertainty expression characteristics of fuzzy systems, medical image data is transformed into fuzzy quantized data, and a fuzzy inference system is established. Experimental verification confirms the method’s effectiveness and accuracy in predicting brain diseases, thereby offering novel insights and methodologies for medical image processing research. Zhang et al.^11^ extensively discussed the applications of artificial intelligence algorithms, including neural networks, random forests, and support vector machines, in image classification, object detection, and image segmentation. These algorithms demonstrate the capability to process large volumes of image data with heightened accuracy and efficiency, consequently improving data processing efficiency and accuracy. Notably, they find substantial utility in the diagnosis and treatment of medical images. Khaleel^12^ primarily explored swarm intelligence-based image compression methods, encompassing ant colony algorithms and particle swarm algorithms, to enhance image transmission efficiency and reduce storage space. These optimization algorithms based on swarm intelligence exhibit significant potential in image processing and play a crucial role in enhancing compression quality while ensuring transmission efficiency. Pezzotti et al.^13^ introduced an adaptive intelligent algorithm for reconstructing low-sampling-rate Magnetic Resonance Imaging (MRI) images. By integrating atmospheric melting, swarm intelligence, and machine learning techniques, this algorithm enhances the traditional compressed sensing theory, consequently elevating the reconstruction quality of MRI images. The methodology holds immense potential in medical image processing and contributes to improving the diagnostic accuracy of low-quality images. Roach et al.^14^ delved into predicting and designing mechanical compression responses in 3D-printed foam substitute materials utilizing computer vision and artificial intelligence algorithms. The authors predicted the performance and application possibilities of foam substitute materials in mechanical and physical domains by studying factors such as microstructure and thermal shrinkage and employing computer simulation and deep learning techniques. Tang et al.^15^ provided an overview of prominent swarm intelligence algorithms, including ant colony algorithms, particle swarm algorithms, artificial bee colony algorithms, and firefly algorithms. They discussed these algorithms’ characteristics, advantages, disadvantages, and applications in solving optimization problems. Notably, these algorithms exhibit significant advantages in different optimization scenarios and display great potential in diverse applications such as logistics planning, neural network design, image processing, and machine learning. Bhardwaj et al.^16^ introduced the application of swarm intelligence and deep learning-based image recognition technology in cancer identification. This approach enhances the accuracy and efficiency of cancer diagnosis by employing deep learning algorithms for feature extraction from medical images and swarm intelligence algorithms for classification. This methodology holds significant importance in medical image processing as it contributes to achieving earlier and more accurate cancer screening and diagnosis, thereby augmenting the effectiveness and success rate of treatments.

Lossless image compression techniques have gained considerable attention and application due to the widespread use of digital images in various fields. Khandwani et al.^17^ extensively reviewed coding algorithms in lossless image compression, including predictive coding, transform coding, dictionary coding, and iterative coding. The article assessed the strengths and applicability of different algorithms and discussed the future prospects of lossless image compression techniques. Kumar et al.^18^ provided an overview of medical image compression techniques’ current status and future trends, encompassing transform coding, waveform coding, and lossless compression. The article compared algorithms based on compression ratio, fidelity, and speed, offering insights into their distinctions and prospects for future development. Uthayakumar et al.^19^ proposed a wireless sensor network image compression scheme that reduces complexity and enhances reliability. This scheme utilizes neighborhood correlation sequence algorithms, integrating spatial domain information and predictive coding techniques to achieve high-quality image compression and transmission. Rahman et al.^20^ evaluated the performance of state-of-the-art lossless static image compression algorithms based on compression ratio, compression speed, and quality. The article summarized the advantages and limitations of various algorithms and discussed future research directions and emerging trends. Liu et al.^21^ presented a lossless image compression algorithm based on adaptive dictionaries and databases, which demonstrated improved efficiency, speed, compression ratio, and fidelity compared to the traditional Huffman coding algorithm. This approach generates optimized Huffman coding tables at an accelerated pace through histogram statistics and analysis, resulting in enhanced compression quality. Tayyeh et al.^22^ introduced an image steganography technique that combines least significant bit (LSB) embedding and Deflate compression. This scheme conceals secret information within the LSBs of an image, enhancing information concealment without compromising image quality. The article elaborated on the principles of LSB steganography and Deflate compression, along with practical scenarios and applications of this technique. In addition to discussing computational/neural network model-based applications for cancer detection and early diagnosis, attention should be given to bone-related studies. These studies highlight the synergy between synchrotron radiation imaging and AI-based models for early injury detection, pioneering the translation of synchrotron radiation results into clinical practice. For instance, Shen et al.^23^ employed CNNs, residual neural networks, and transfer learning to classify and predict the mechanical state of cortical and trabecular bone tissues. Through an optimized CNN architecture, they developed a training model for classifying new images on cortical and trabecular bone, demonstrating the potential to develop models targeting high-resolution SR-microCT images, even with limited training samples. Further development with more data and training methods could yield novel, fundamental, and machine learning-driven insights into bone microstructures. Buccino and Aiazzi et al.^24^ integrated micro-crack propagation visualization with CNNs. Their AI tool, based on a large number of human synchrotron images from healthy and osteoporotic femoral heads tested for micro-compression, automatically detected lacunae and micro-cracks at various scales, marking a significant advancement in bone research.

In conclusion, lossless image compression techniques have found widespread application and garnered significant attention across various domains. By comparing coding algorithms in terms of compression ratio, fidelity, and speed, suitable compression techniques can be selected for specific scenarios. Moreover, as the utilization of digital images continues to expand, lossless image compression techniques undergo constant innovation and optimization, better meeting practical application requirements and providing new ideas and directions for future research.

### Recent studies on 3D image compression algorithms and NX model compression algorithms

In recent years, the widespread adoption of 3D imaging technology has revolutionized various fields, including material science, biomedical science, and mechanical engineering. Alqadami et al.^25^ introduced a flexible electromagnetic helmet for non-invasive 3D electromagnetic head imaging detection. This device, constructed using isotropic materials, generates a 3D electromagnetic field on the head’s surface, enabling brain imaging diagnosis through potential distribution measurements. Narazaki et al.^26^ presented a 3D displacement measurement algorithm based on physical and graphical models. By establishing accurate physical models and leveraging computer vision and image processing techniques, this approach facilitates functions such as 3D object deformation monitoring, displacement tracking, and morphological analysis, offering extensive application potential. Florkow et al.^27^ provided an in-depth comparison of MRI and Computed Tomography (CT) for 3D skeletal imaging. The article analyzed the advantages, disadvantages, imaging principles, and current application status of MRI and CT. It also discussed the prospects of these techniques in the diagnosis and treatment planning of orthopedic diseases.

As various imaging technologies continue to evolve, reducing costs and time consumption while ensuring imaging quality has become a significant research focus. In response, scholars and researchers have proposed diverse methods and techniques. Yuan et al.^28^ introduced the theory, algorithms, and applications of snapshot compressive imaging. This approach leverages sparse representation theory and compressive sensing algorithms to acquire and compress images simultaneously in both time and space domains. It enables the acquisition of high-quality images with reduced costs and increased efficiency. Qiao et al.^29^ proposed a novel synchronous coherent imaging method called “snapshot coherent tomographic imaging.” This technique combines snapshot compressive imaging with coherent tomographic imaging, enabling high-speed, high-resolution, and cost-effective 3D imaging. Its potential applications span fields such as medicine and biology. Wang et al.^30^ introduced a joint range migration and sparse reconstruction network for 3D millimeter-wave imaging. This network design incorporates a reconstruction encoder and weighted fully connected layers, which possess powerful representation learning capabilities. It effectively enhances imaging quality while demonstrating stability and real-time performance. Chaithya et al.^31^ presented a fully 3D “Sparkling” magnetic resonance imaging (MRI) technique. This technique achieves high-resolution imaging in a rapid and compact scan by combining optimized gradient waveforms and waveform trajectories. The study also proposed a novel experimental design for ablation, demonstrating the effectiveness and practicality of the technique.

In conclusion, ongoing advancements in imaging technologies have led to the proposal of various methods and techniques that address imaging quality, cost, and time consumption. These innovations bring about more convenient applications, and imaging technologies will continue to play crucial roles in driving future technological revolutions and industrial upgrading.

## Design and specific application of the DEFLATE algorithm in NX 3D image model compression

### Design and analysis of the DEFLATE algorithm

**Figure 1:**
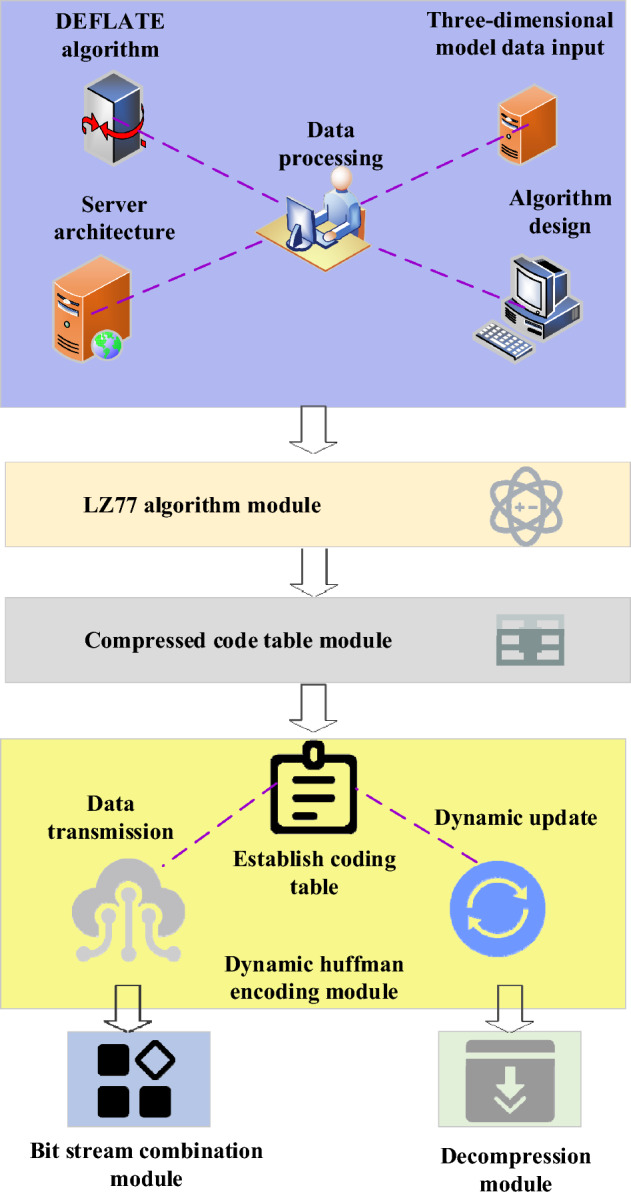
Structural framework of the design and analysis of the DEFLATE algorithm (Source: Visio 2013 software and https://www.iconfont.cn/).

The DEFLATE algorithm is a widely utilized data compression algorithm and serves as the foundation for substantial optimization efforts. Its design incorporates key components such as the compression code table, dynamic Huffman coding technique, LZ77 algorithm, and bitstream combination technique^32^. The compression code table, generated through data analysis and statistical information, plays a critical role in the compression process. The dynamic Huffman coding technique dynamically generates the encoding table during compression to adapt to the data’s compression requirements. The LZ77 algorithm detects and replaces duplicate data, effectively reducing data volume. The design and analysis of the DEFLATE algorithm follow the structural framework illustrated in Fig. 1.

### Characteristics and compression requirements analysis of NX 3D image models

NX 3D image models possess notable characteristics, including high resolution and a high signal-to-noise ratio. Consequently, they require substantial storage space during the image acquisition and storage processes. Current compression algorithms for NX 3D image models can be classified into two categories: a combination of traditional compressive sensing and reconstruction methods with data-driven approaches and solely data-driven methods^33,34^. These approaches exhibit excellent interpretability and adaptability when compressing NX 3D image models, allowing for rapid signal recovery. Furthermore, they significantly reduce the storage space required during compression, facilitating subsequent image processing and analysis tasks.

Due to the multidimensional nature of NX 3D image models and variations in pixel values and quantities across different resolutions, different compression algorithms and parameters need to be applied at different image levels. Among the compression algorithms, the DEFLATE algorithm is widely employed for image compression due to its high compression ratio, fast processing speed, and compact compressed file size. Combining the DEFLATE algorithm with deep learning-based compressive sensing algorithms further enhances the efficiency and accuracy of the compression and reconstruction processes.

**Figure 2:**
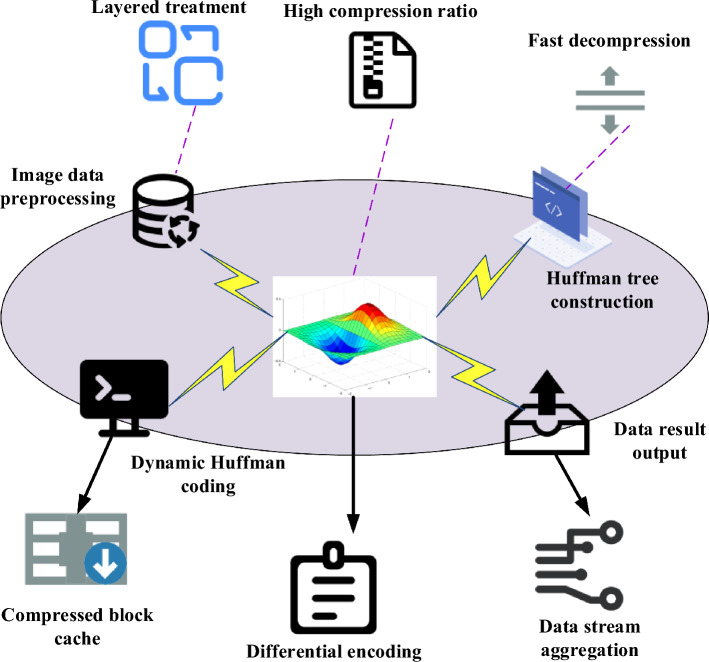
Structure diagram depicting the characteristics of NX 3D image models and data compression requirements (Source: Visio 2013 software and https://www.iconfont.cn/).

Figure 2 illustrates the analysis of NX 3D image model characteristics and the specific requirements for data compression.

### Design of the optimized DEFLATE algorithm

In order to enhance the performance of the DEFLATE algorithm in compressing NX 3D image models, this study conducts optimizations in two key components: the LZ77 algorithm and Huffman coding. In the traditional LZ77 algorithm, a sliding window is typically employed to search for repeated substrings. This study proposes a dynamic window-based matching algorithm to expedite the discovery of matches. The specific matching rules are illustrated in Eq. (1):

1 $$\text{Maximum Match Length}=\text{max}(\text{ML}-\text{HT},3)$$

In Eq. (1), ML represents the current length of the matched duplicate substring, and HT denotes the heuristic threshold. This threshold can be dynamically adjusted based on the characteristics of the actual dataset. If ML—HT is less than 3, the experiment sets the matching length to 3 to ensure a minimum matching length of 3. While maintaining compression ratios, this approach significantly enhances matching speed and accuracy. The traditional LZ77 algorithm employs a fixed-size sliding window, which may potentially miss matches as the window slides. This study introduces the concept of a dynamic window. The calculation of the dynamic window is shown in Eq. (2):

2 $$\text{Window Size}=\text{min}(\text{MWS},\text{CP}-\text{FMP}+\text{HI})$$

In Eq. (2), MWS represents the maximum allowable size of the window. CP denotes the current processing position. FMP signifies the furthest matching position before the current position. HI represents the heuristic increment, which is dynamically adjusted based on the dataset’s characteristics. By dynamically adjusting the window size, the experiment can better capture potential duplicate substrings, thereby enhancing matching accuracy. Through these improvements, the LZ77 algorithm efficiently identifies and utilizes duplicate substrings in the DEFLATE compression process, consequently improving compression performance. These enhancements simultaneously maintain compression ratios, making the algorithm more practical in real-world applications.

Huffman coding plays a critical role in determining symbol encoding lengths within the DEFLATE algorithm. This study optimized Huffman coding to enhance its performance. The experiment introduced the concept of an adaptive coding tree, enabling the coding tree to dynamically adjust based on input data distribution, thus better accommodating various data types. Traditional Huffman coding constructs the coding tree based on data frequencies. The optimization process introduced adaptability, allowing the coding tree to automatically adjust based on the current input data during the encoding process. This adaptability effectively caters to the characteristics of different datasets, improving encoding efficiency.

In order to minimize the average encoding length, the experiment introduced a restriction on the minimum encoding length. In traditional Huffman coding, the encoding length for each symbol is variable. This study restricted the minimum encoding length, ensuring even symbols with lower frequencies have a reasonable encoding length. This constraint helps reduce the overall encoding length and improve compression ratios. Huffman coding is depicted in Eq. (3):

3 $$\text{Code Length}=\text{max}(\text{Minimum Code Length},\text{Original Code Length})$$

In Eq. (3), the Minimum Code Length is an introduced constraint, and the Original Code Length is calculated based on the traditional Huffman coding method. By comparing the Minimum Code Length with the Original Code Length, the experiment selects the larger of the two as the final encoding length. This approach ensures that even symbols with lower frequencies have a reasonable encoding length, thereby improving compression efficiency. Through the aforementioned optimizations, this study enhances the performance of Huffman coding, making it more suitable for various types of datasets and improving the overall compression performance of the DEFLATE algorithm.

**Figure 3:**
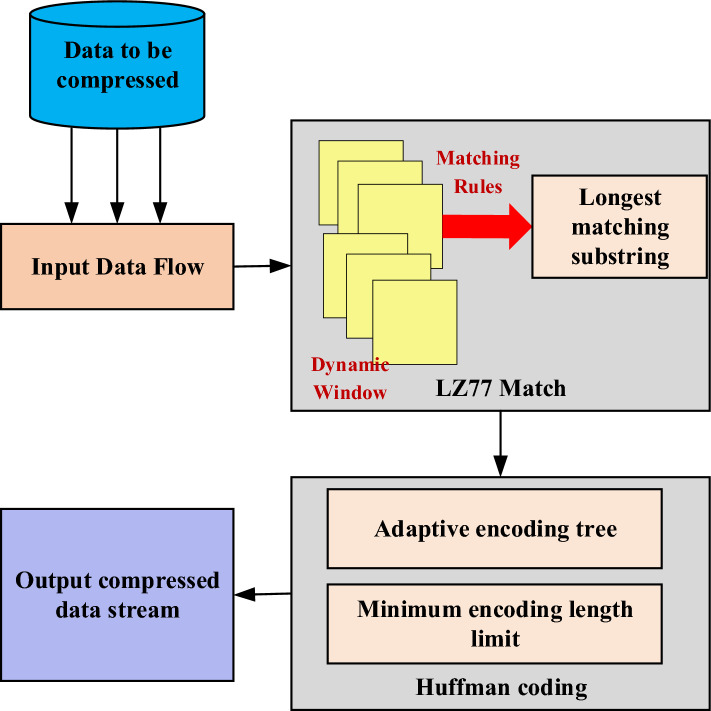
Schematic of the optimized DEFLATE algorithm.

In order to illustrate the proposed optimization strategies, Fig. 3 depicts the workflow of the improved DEFLATE algorithm:

Figure 3 showcases the workflow of the proposed optimized DEFLATE algorithm, consisting of two primary phases: the LZ77 matching phase and the Huffman coding phase. The LZ77 matching phase demonstrates the operation of the dynamic window, with the window size continuously adjusting based on dynamic rules to accommodate matching requirements at different positions. The algorithm identifies the longest matching substring, a crucial step in the LZ77 algorithm. The Huffman coding phase highlights the application of the adaptive coding tree and the use of minimum encoding lengths. Each symbol is mapped to its corresponding code, ensuring compact encoding. By imposing a constraint on the minimum encoding length, this study avoids the issue of excessively short encoding lengths in extreme cases, thereby improving compression efficiency.

### Experimental design

This study collects production data comprising NX 3D image models with multiple resolution levels to assess the optimized DEFLATE algorithm’s effectiveness in compressing NX 3D image models. The production data of NX 3D image models encompass images at various resolution levels, often depicted stereoscopically, capturing object or scene details and structures across different spatial dimensions. These datasets typically span engineering, medical, or scientific domains, serving digital modeling, simulation, analysis, and visualization purposes. The dataset comprises images categorized into the following resolution tiers: (1) Low resolution: These images offer limited spatial detail and clarity, primarily for quick previews or overall shape identification, such as models used in initial design phases or rapid prototyping. (2) Medium resolution: These images exhibit enhanced clarity and detail compared to low-resolution counterparts, suitable for detailed design, engineering analysis, and simulation, including models utilized in product design and manufacturing for structural analysis, assembly, and material property assessments. (3) High resolution: These images feature the utmost spatial detail and clarity, ideal for precise modeling, precision manufacturing, and quality control, such as models employed in medical imaging for anatomical studies or clinical diagnosis. Each image at every resolution level is stored in the NX file format, containing comprehensive information about image geometry, material, texture, and color. Additionally, metadata related to the images, such as acquisition device details, sampling rate, coordinate system, and measurement units, may be included. These high-resolution image models, characterized by their signal-to-noise ratio, demand substantial storage space during acquisition and storage. To address this, compression techniques like the DEFLATE algorithm, Wavelet algorithm, and C-Bone algorithm are employed. Experimental analysis involves compressing images of varied sizes, evaluating compression ratios, and assessing compression times. Different compression algorithms and parameters are employed to compress images at various levels^35^. For the highest-resolution image layer, a compression-level parameter of 5, a window-bits parameter of 13, and a hash-bits parameter of 12 are utilized, along with appropriate compression offsets. For lower-resolution image layers, lower compression-level parameters and smaller window-bits and hash-bits parameters are employed to avoid excessive compression time and unnecessary impact on image quality.

Furthermore, for performance assessment, the proposed DEFLATE algorithm for 3D image compression is evaluated in terms of compression ratio, data throughput rate, data transmission accuracy, and data transmission reliability in comparison with the Wavelet algorithm and C-Bone algorithm. Furthermore, the optimized DEFLATE algorithm’s performance is compared to wavelet algorithms, the C-Bone algorithm, the Lempel–Ziv–Welch algorithm, the arithmetic coding algorithm, and the Burrows-Wheeler transform algorithm. In the process of experimental statistics and data analysis, the following notations are used: Swavelet algorithm denotes the implementation of the Wavelet algorithm for small-scale models; SCBone algorithm represents the implementation of the C-Bone algorithm for small-scale models; SDEFLATE algorithm refers to the implementation of the DEFLATE algorithm for small-scale models. Similarly, MDEFLATE algorithm denotes the implementation of the DEFLATE algorithm for medium-scale models; Mwavelet algorithm represents the implementation of the Wavelet algorithm for medium-scale models; Lwavelet algorithm represents the implementation of the Wavelet algorithm for large-scale models; LlempelZivWelch indicates the implementation of the Lempel–Ziv-Welch algorithm for large-scale models; LDEFLATE algorithm signifies the implementation of the DEFLATE algorithm for large-scale models.

## Result and discussion

### Performance comparison of different data compression and transmission algorithms in small and medium-scale models

**Figure 4:**
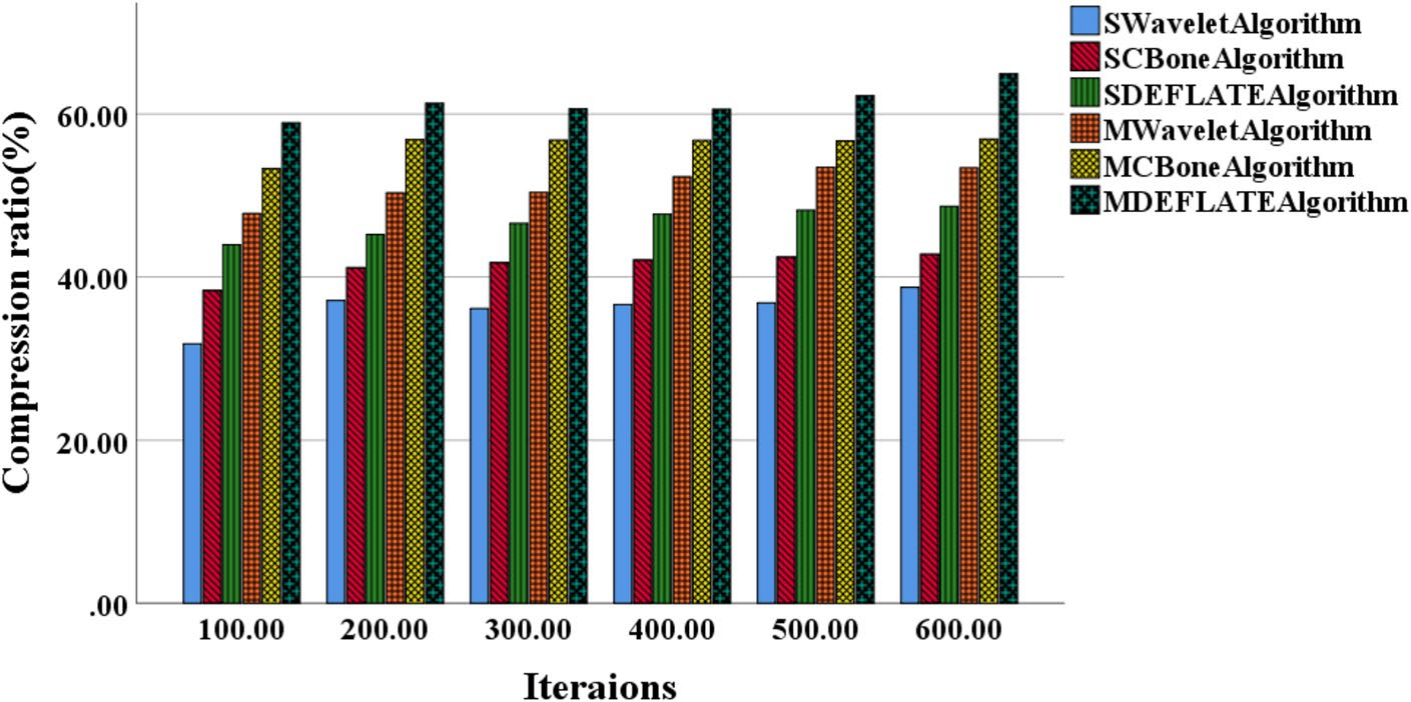
Variation curves of data compression ratios for small and medium-scale models using different algorithms.

**Figure 5:**
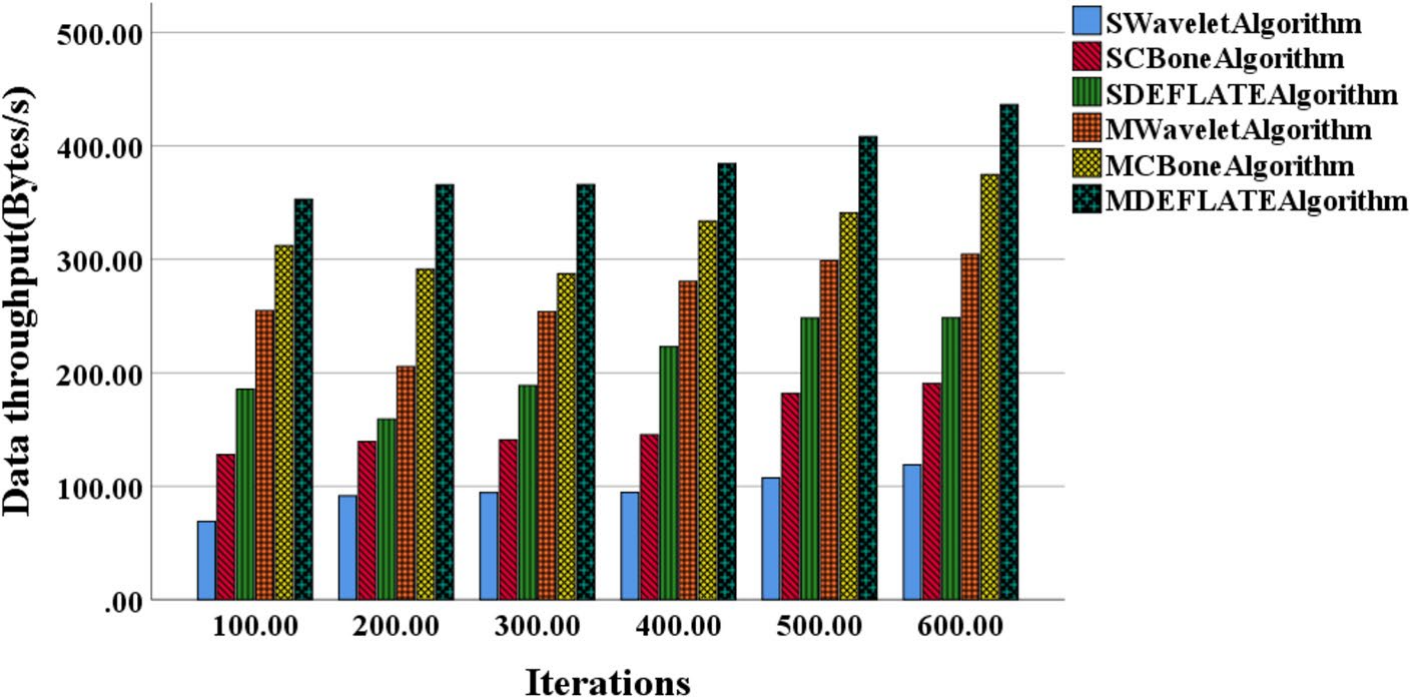
Variation curves of model data throughput using different algorithms for small and medium-scale models.

This study aims to compare the performance of various algorithms for data compression and transmission in small and medium-scale models and explore optimal algorithm selection and performance optimization strategies. Figure 4 illustrates the variation curves of data compression ratios for small and medium-scale models using different algorithms. Figure 5 presents the performance variation curves of these algorithms in terms of model data throughput. Table 1 presents the variation in compression ratios and throughput as the number of iterations increases for different algorithms.

**Table 1:** The changes in compression ratios and throughput as the number of iterations increases for different algorithms.

Number of iterations	Compression rate of MDEFLATE algorithm (%)	Compression rate of Wavelet algorithm (%)	The data throughput rate of MDEFLATE algorithm (MB/s)	The data throughput rate of the Wavelet algorithm (MB/s)
500	52.5%	45.6%	74.6	50.2
600	54.8%	47.1%	77.2	51.6
700	56.2%	48.5%	79.8	53.7
800	57.9%	49.8%	82.5	55.9

Analysis of Fig. 4 reveals an overall increasing trend in data compression ratios for small and medium-scale models across different algorithms. The SWavelet algorithm, SCBone algorithm, and SDEFLATE algorithm exhibit similar growth trends in data compression ratios, with the SWavelet algorithm demonstrating a slightly slower growth rate compared to the other two algorithms. With an increase in the number of iterations, all algorithms show improved data compression ratios, particularly notable in the case of the MWavelet algorithm, MCBone algorithm, and MDEFLATE algorithm. Notably, the MDEFLATE algorithm achieves higher compression ratios, especially at higher iteration counts, compared to the Wavelet algorithm.

In Table 1, as the number of iterations increases, the MDEFLATE algorithm consistently demonstrates an improving trend in compression ratios compared to the Wavelet algorithm. When the number of iterations reaches 800, the MDEFLATE algorithm achieves a compression ratio of 57.9%, while the Wavelet algorithm only reaches 49.8%. This data indicates that the MDEFLATE algorithm can compress data to a smaller size for the same data volume, offering more storage space. Moreover, the MDEFLATE algorithm exhibits significant improvements in compression ratios and excels in data throughput. At 800 iterations, the MDEFLATE algorithm achieves a data throughput of 82.5 MB/s, which is approximately 49% higher than the 55.9 MB/s achieved by the Wavelet algorithm. The data means that during data transmission, the MDEFLATE algorithm can process and transmit data more rapidly, enhancing data transfer speed and efficiency. Furthermore, in terms of stability and reliability, the MDEFLATE algorithm maintains consistent performance across different numbers of iterations, while the performance of the Wavelet algorithm tends to be more fluctuating. This suggests that the MDEFLATE algorithm exhibits better stability and reliability in long-term data transmission, ensuring continuous, efficient data compression and transmission services.

In summary, the optimized DEFLATE algorithm (MDEFLATE algorithm) achieves an additional 15% compression ratio and a 49% improvement in data throughput under high iteration counts while maintaining stable performance. These advantages make the MDEFLATEA algorithm excel in handling the data transmission of large-scale 3D image models, providing efficient, stable, and reliable support for practical applications.

Figure 5 demonstrates that, in small-scale models, the SWavelet algorithm exhibits the lowest data throughput, while the MDEFLATE algorithm achieves the highest data throughput. In medium-scale models, the SCBone algorithm displays the lowest data throughput, while the MDEFLATEA algorithm remains superior in terms of data throughput. As the number of iterations increases, the data throughput of each algorithm exhibits an upward trend. Specifically, the MDEFLATE algorithm achieves the highest data throughput in small-scale models, whereas in medium-scale models, it continues to outperform other algorithms, maintaining the highest data throughput.

**Figure 6:**
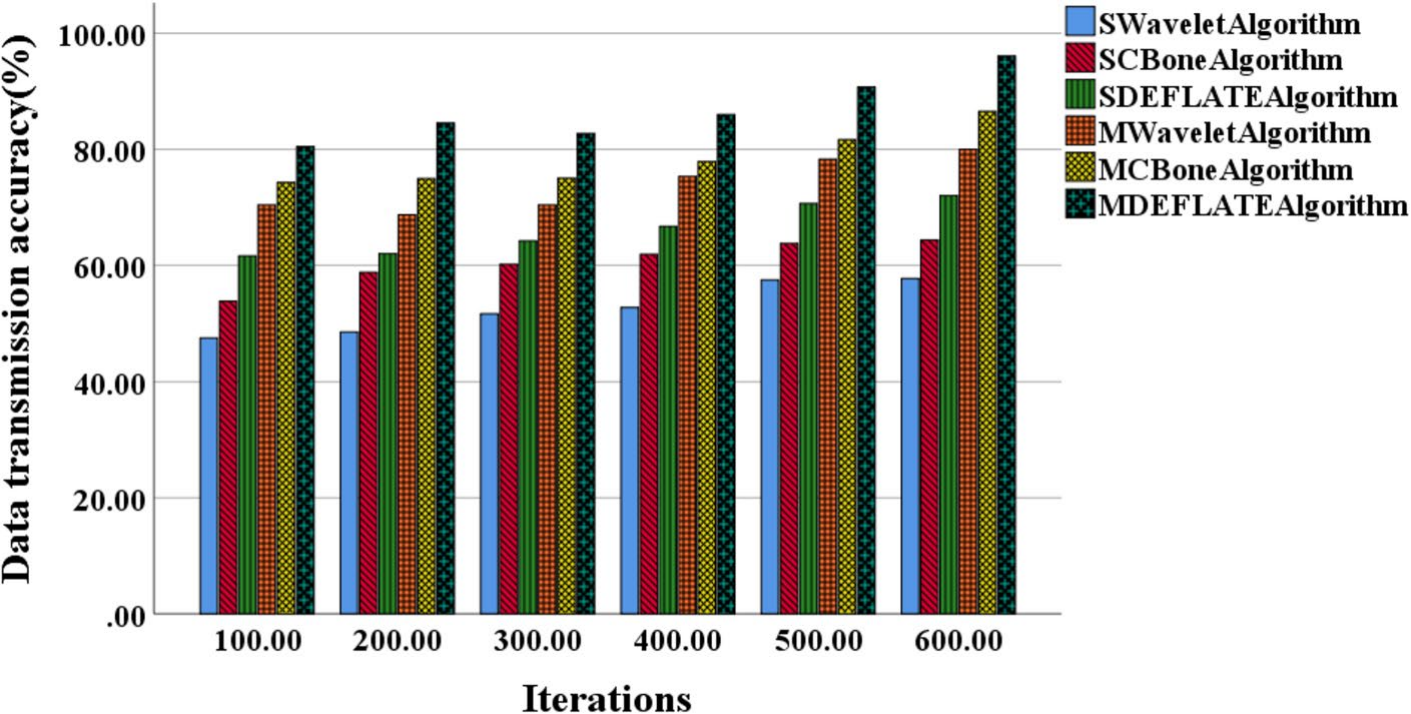
Variation curves of model data transmission accuracy using different algorithms for small and medium-scale models.

**Figure 7:**
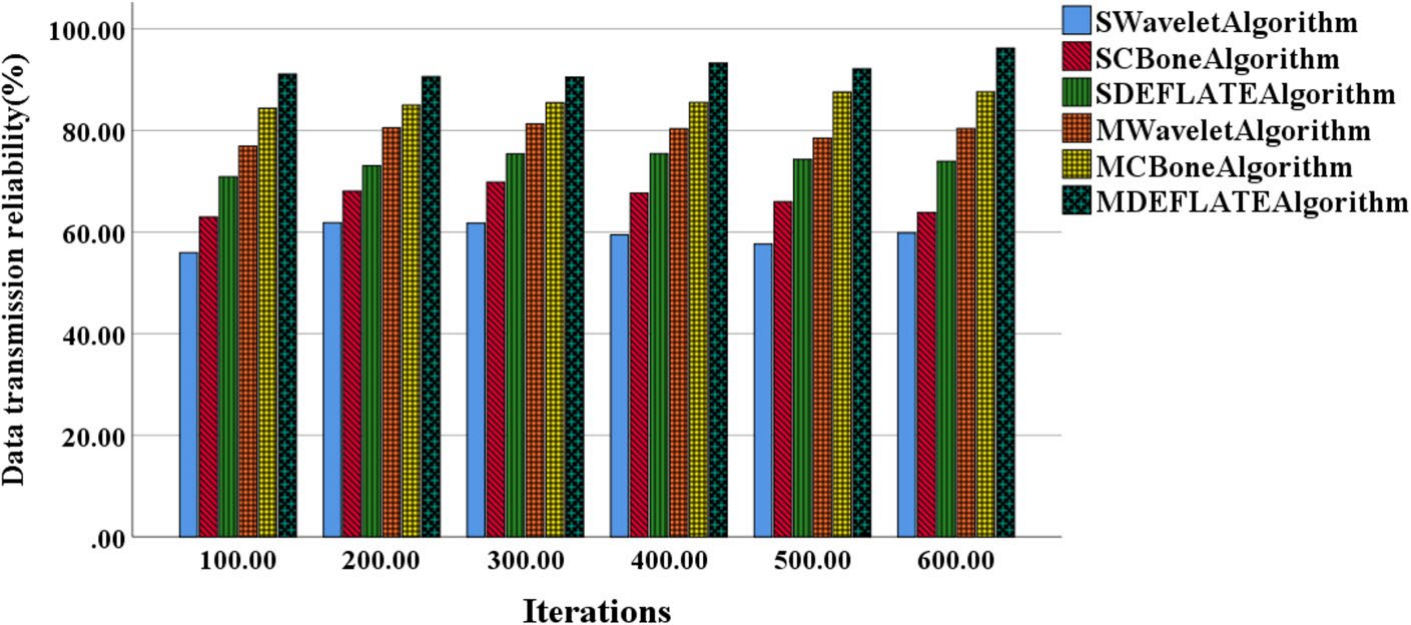
Variation curves of model data transmission reliability using different algorithms for small and medium-scale models.

Additionally, Figs. 6 and 7 illustrate the performance of different algorithms in terms of data transmission accuracy and reliability.

Figure 6 reveals that the SDEFLATE algorithm consistently achieves the highest transmission accuracy in small-scale models, while the SWavelet algorithm exhibits the lowest transmission accuracy. The MWavelet algorithm demonstrates the lowest transmission accuracy in medium-scale models, whereas the MDEFLATE algorithm achieves the highest transmission accuracy. As the number of iterations increases, the differences in transmission accuracy among different algorithms gradually diminish, although significant variations still exist. At 100 iterations, the transmission accuracy based on the SDEFLATE algorithm is 61.6%, while the MWavelet algorithm already reaches 70.4%. At 600 iterations, the MDEFLATE algorithm achieves a transmission accuracy of 96.1%, while the SCBone algorithm only attains 72.0% transmission accuracy.

Figure 7 demonstrates that the MCBone and MDEFLATE algorithms consistently exhibit higher transmission reliability, while the SCBone and SWavelet algorithms display lower transmission reliability. In small-scale models, as the number of iterations increases, the SCBone and SWavelet algorithms show slow transmission reliability growth. In medium-scale models, MWavelet and MDEFLATE algorithms exhibit relatively larger variations in transmission reliability. Overall, the DEFLATE algorithm demonstrates relatively stable performance in small and medium-scale models, ranking first in transmission reliability. Conversely, Wavelet algorithm generally exhibits lower transmission reliability in small and medium-scale models.

### Performance comparison of different data compression and transfer algorithms in large-scale models

**Figure 8:**
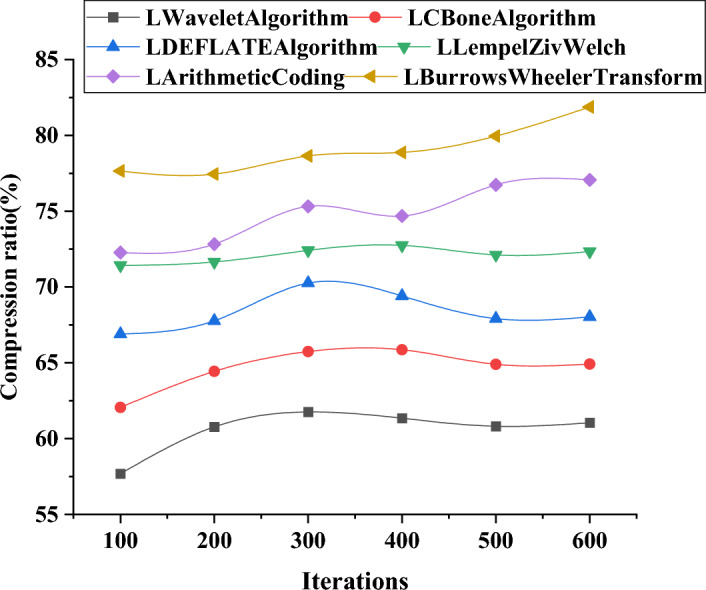
Variation curve of data compression ratios using different algorithms in large-scale models.

Furthermore, an investigation is conducted to assess the performance of different data compression and transmission algorithms in large-scale models by comparing their data compression and transmission effectiveness with those of small and medium-scale models. Figure 8 illustrates the variation curves of data compression ratios using different algorithms in large-scale models. Figure 8 presents the variation curves of model data throughput with respect to the number of iterations.

Figure 8 demonstrates that as the data scale increases, the effectiveness of data compression using different algorithms improves. This upward trend may stabilize or decline after reaching a certain number of iterations. At 100 iterations, the LBurrowsWheelerTransform algorithm achieves a higher compression ratio compared to other algorithms, while the LSDEFLATE algorithm exhibits a lower compression ratio. As the number of iterations increases, the LWavelet algorithm and LCBone algorithm consistently increase compression ratios, reaching higher levels at 600 iterations. At 600 iterations, the LBurrows Wheeler Transform algorithm attains the highest compression ratio, while the LWavelet algorithm and LCBone algorithm rank in the middle. The LLempelZivWelch algorithm and the LArithmeticCoding algorithm also achieve relatively high compression ratios.

**Figure 9:**
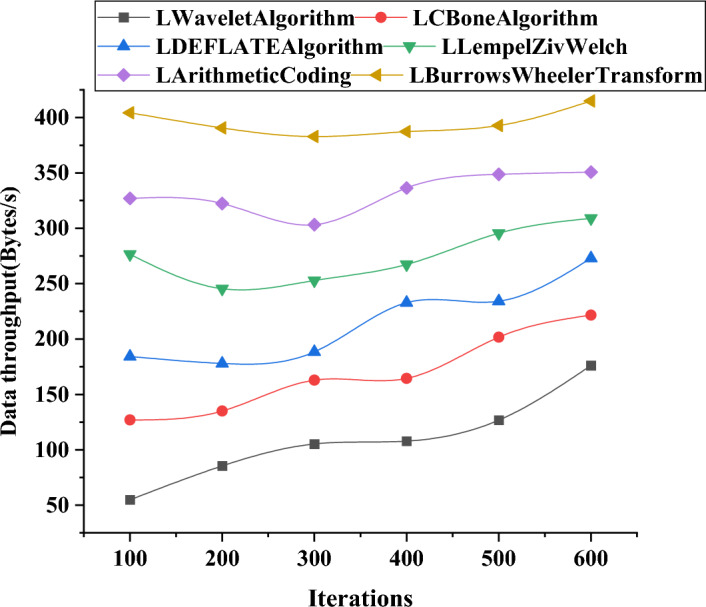
Variation curve of model data throughput using different algorithms in large-scale models.

Figure 9 shows that at 100 iterations, the throughput of the LWavelet algorithm is approximately 54.89 Bytes/s, which increases to approximately 176.09 Bytes/s at 600 iterations. Overall, the throughput improves by approximately threefold. LArithmeticCoding achieves a throughput of approximately 327.01 Bytes/s at 100 iterations, increasing to approximately 350.72 Bytes/s at 600 iterations. The LBurrowsWheelerTransform algorithm exhibits a generally stable upward trend. At 100 iterations, the throughput is approximately 404.29 Bytes/s, and at 600 iterations, it reaches approximately 414.89 Bytes/s. The increase in throughput for this algorithm is relatively small.

**Figure 10:**
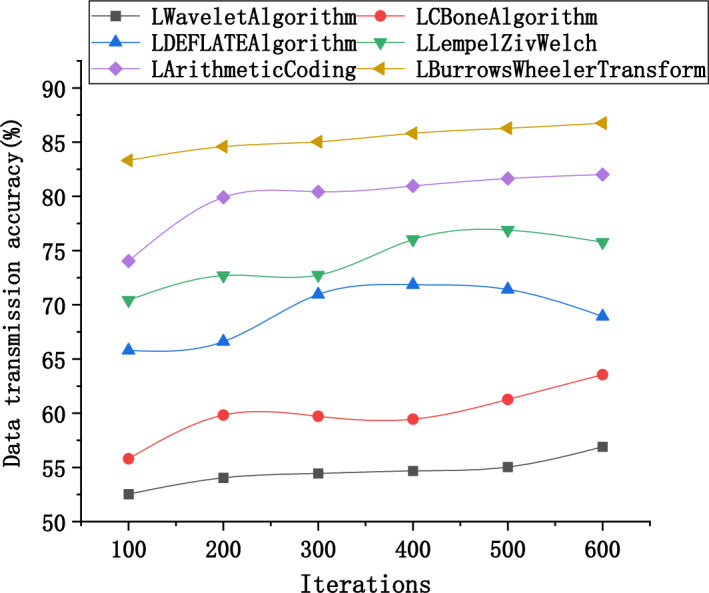
Variation curves of data transmission accuracy using different algorithms in large-scale models.

**Figure 11:**
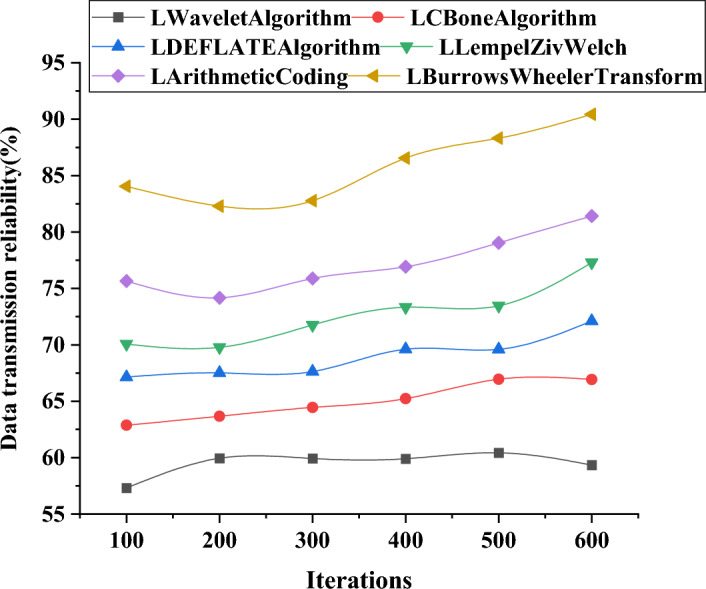
Variation curves of data transmission reliability using different algorithms in large-scale models.

Furthermore, detailed performance comparisons of these algorithms in terms of data transmission accuracy and reliability are presented in Figs. 10 and 11.

Figure 10 illustrates the transmission accuracy of six different compression algorithms (LWavelet algorithm, LCBone algorithm, LDEFLATE algorithm, LLempelZivWelch, LArithmeticCoding, LBurrows Wheeler Transform) under varying numbers of iterations. At the same number of iterations, the transmission accuracy of each algorithm shows little difference. However, with increasing iterations, these differences gradually expand and stabilize. During large-scale data transmission, the optimized DEFLATE algorithm (LDEFLATE algorithm) excels when there are a large number of iterations. Compared to other algorithms, the LDEFLATE algorithm exhibits a trend of gradually improving transmission accuracy with an increase in iterations, while the improvement rate of other algorithms is relatively slower. This highlights the outstanding performance of the optimized DEFLATE algorithm in handling the transmission of large-scale 3D image models, with its transmission accuracy not only being excellent initially but also remaining stable during long-term operation, confirming its exceptional performance in practical applications.

In Fig. 11, under varying numbers of iterations, the data transmission reliability of six different compression algorithms (LWavelet algorithm, LCBone algorithm, LDEFLATE algorithm, LLempelZivWelch, LArithmetic Coding, LBurrows Wheeler Transform) demonstrates a slow and stable growth trend. Concerning data transmission reliability, the LDEFLATE algorithm exhibits outstanding performance as the number of iterations increases. Compared to other algorithms, the optimized DEFLATE algorithm (LDEFLATE algorithm) and the LBurrowsWheelerTransform algorithm display similar slow and continuous growth trends, which may be attributed to their robust algorithm principles and implementation methods. In contrast, the performance of other algorithms in terms of data transmission reliability tends to be relatively unstable. For instance, the LWavelet algorithm exhibits a decreasing trend before 600 iterations and a slow increase afterward. LCBone algorithm, after an initial increase, experiences a decline in data transmission reliability, which may be related to algorithm design and implementation. LLempelZivWelch algorithm shows a decline followed by an increase, possibly due to its data compression approach. These trends emphasize the stability and reliability of the optimized DEFLATE algorithm in long-term data transmission, confirming its exceptional performance in the transmission of large-scale 3D image models.

The study conducts experiments using 3D image data acquired through synchrotron radiation technology to evaluate and compare the compression efficacy of the DEFLATE algorithm for such specialized data. Beyond assessing compression ratios, the study also analyzes the time required for the compression process to provide a comprehensive evaluation of the algorithm’s performance. The experimental dataset comprises high-resolution 3D images obtained from synchrotron facilities, known for their exceptionally high signal-to-noise ratio and resolution, making them valuable for scientific research and industrial inspection. Given the substantial volumes of synchrotron radiation image data, optimized compression algorithms are crucial for conserving storage space and improving data processing efficiency. The experimental setup selects synchrotron 3D image data of varying scales, including small, medium, and large images. Alongside the DEFLATE algorithm, several other common compression algorithms, such as the Wavelet algorithm and C-Bone algorithm, are utilized to compress synchrotron radiation images. The compression performance of synchrotron 3D images under different algorithms is summarized in Table 2.

**Table 2:** Compression performance of synchrotron 3D images under different algorithms.

Iterations	DEFLATE Algorithm Compression Ratio (%)	DEFLATE Algorithm Compression Time (s)	Wavelet Algorithm Compression Ratio (%)	Wavelet Algorithm Compression Time (s)
500	53.7	21.8	46.9	19.2
600	55.4	23.5	48.2	20.6
700	56.9	25.1	49.5	22.1
800	58.3	26.7	50.9	23.5

Table 2 illustrates the compression performance of synchrotron 3D images under different algorithms. It is evident that, at the same number of iterations, the DEFLATE algorithm generally achieves a higher compression ratio compared to the Wavelet algorithm, albeit with slightly longer compression times. As the number of iterations increases, the compression ratio of the DEFLATE algorithm demonstrates a stable upward trend, with a relatively gradual increase in compression time, indicating the effectiveness and efficiency of the DEFLATE algorithm in processing complex data. This study offers an insightful analysis of the compression performance of synchrotron 3D images, showcasing the feasibility and superiority of the DEFLATE algorithm in compressing such high-resolution image data. Further optimization of the DEFLATE algorithm is anticipated to significantly contribute to future scientific research and industrial applications.

## Conclusion

This study aims to enhance the compression performance of 3D image models by optimizing and designing the DEFLATE algorithm, specifically focusing on reducing the compression time. The study conducts a series of experiments on compressing 3D image models in NX, involving algorithm design and detailed theoretical analysis. Experimental objects include 3D models of different scales, and various compression algorithms are compared. The optimized DEFLATE algorithm performs well in compressing small and medium-sized 3D models. Specifically, it achieves a compression rate improvement of 15% and 49% compared to the Wavelet and C-Bone algorithms, respectively, while demonstrating shorter compression times. Regarding large-scale 3D models, although the compression rate of the DEFLATE algorithm is comparable to other algorithms, its compression time is significantly reduced, and it improves data transmission reliability by 12.1%. Furthermore, this study addresses the requirement for multi-level perceptual compression by employing different compression algorithms and parameters for distinct image levels. A scientific and standardized workflow for compressing 3D image models is proposed. However, certain limitations exist in this study. Multiple compression algorithms were compared; however, there was a lack of analysis regarding the specific performance of optimization and design strategies for the DEFLATE algorithm. These strategies include compression code tables, dynamic Huffman coding techniques, LZ77 algorithm, and bitstream combination techniques. Future research should incorporate more experimental data to further elaborate on the DEFLATE algorithm’s optimization strategies and improve the models’ data transmission accuracy.

### Supplementary Information

Supplementary Information 1.

Supplementary Information 2.

## Data Availability

All data generated or analysed during this study are included in this published article [and its Supplementary Information files].
